# Stereoscopic visual stimuli for examining biological motion perception and unanticipated steering manoeuvres in people with Parkinson's disease

**DOI:** 10.1016/j.mex.2021.101350

**Published:** 2021-04-15

**Authors:** Stacy W.L. Foo

**Affiliations:** School of Human Sciences, The University of Western Australia, Perth, Australia; Research Administration, National Cancer Centre Singapore, Singapore

**Keywords:** Biological motion, Ecological validity, Stereoscopic cinematography, Stereoscopic display, Visual perception

## Abstract

Community falls in people with Parkinson's disease (PwPD) are common, costly, and often unanticipated. Aside from static obstacles, it has been reported that oncoming people in community settings pose problems for PwPD when navigating. This suggests that PwPD may have difficulty (i) perceiving biological motion and action possibilities, and (ii) steering out of the way of oncoming persons. To date, laboratory research that investigated unanticipated steering manoeuvres in PwPD have only incorporated light- or arrow-based visual stimuli to simulate the spatiotemporal demands of these movements. However, such simple stimuli are not ecologically valid for examining biological motion perception and unanticipated steering manoeuvres used in avoiding oncoming people. To improve the generalisability of laboratory research in this field, a set of stereoscopic visual stimuli that feature an oncoming person initiating a sudden change in direction was developed for PwPD to engage with. Specifically, we modified and improved existing cinematographic techniques, software, and stereoscopic display technology to bring about:

• Ambulatory scenarios that were quasi-immersed with the laboratory environment.
• Enhanced realism.
• Better temporal consistency in video playback.

## Graphical abstract

**Figure:**
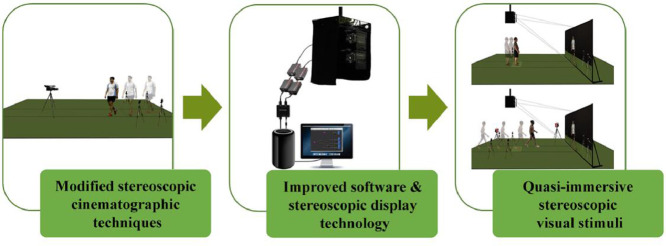

##  

Specifications Table

**Table:** 

Subject Area:	Engineering
More specific subject area:	*Stereoscopic Cinematography* *Display Technology*
Method name:	*Stereoscopic cinematography for creating quasi-immersive ambulatory scenes in laboratory environment*
Name and reference of original method:	*Lee, M. J. C., Bourke, P., Alderson, J. A., Lloyd, D. G., & Lay, B. (2010). Stereoscopic filming for investigating evasive side-stepping and anterior cruciate ligament injury risk. Paper presented at the Stereoscopic Displays and Applications XXI, San Jose, CA, USA.*
Resource availability:	http://paulbourke.net/stereographics/

## *Method details

### Background

Community ambulation reflects an individual's general well-being and independence [1]. However, this ability is often compromised in people with Parkinson's disease (PwPD) especially in crowded environments [2,3]. In community settings, Stack and Roberts [4] previously reported that some PwPD recounted falling incidents due to abrupt changes in direction from avoiding oncoming people. As moving people are ubiquitous in crowded environments such as walkways and malls, changes in direction while walking are often unanticipated [2]. To simulate the spatiotemporal demands of unanticipated directional changes within laboratory settings, some researchers have incorporated light- or arrow-based visual stimuli to objectively examine the steering mechanics in PwPD [5], [6], [7]. Although much insight has been gained, such simple visual stimuli disregard the underlying cognitive mechanisms (e.g., perception of biological motion and action possibilities, attention etc.) that contribute towards the production of the steering manoeuvres used in avoiding oncoming people. Apart from impairment in steering mechanics [5], [6], [7], the inability to avoid oncoming people [4] suggests that PwPD may have difficulty perceiving biological motion and the possibilities for action under those circumstances.

To push forth the boundaries of laboratory research in examining biological motion perception and unanticipated steering mechanics in PwPD, we propose the use of stereoscopic three-dimensional (S3D) cinematography and display technology in creating a series of visual stimuli that attempt to encapsulate the realism of real-world ambulatory scenarios. This filming technique has previously been introduced by Lee et al. [8] who developed a series of S3D projections featuring semi-realistic game scenarios for investigating the associations between evasive side-stepping and the risk of anterior cruciate ligament injuries in soccer players [9]. Compared with two-dimensional videos of the same scenes, previous research using eye-tracking [10] and functional brain imaging [11], [12], [13], [14] suggest that the additional depth cues present in S3D videos significantly modulates both dorsal and ventral visual information processing streams that subserve vision-for-action and vision-for-perception [15]. To the best of the author's knowledge, the techniques and technology have yet been used beyond research in sport. Here, this methods paper presents the modifications and improvements made to Lee et al.’s [8] cinematography techniques, software, and display technology to project semi-realistic, S3D ambulatory scenarios that are quasi-immersed with the laboratory environment for examining biological motion perception and unanticipated steering when avoiding an oncoming person for PwPD.

### Filming scenarios and geometrical considerations

**Fig. 1:**
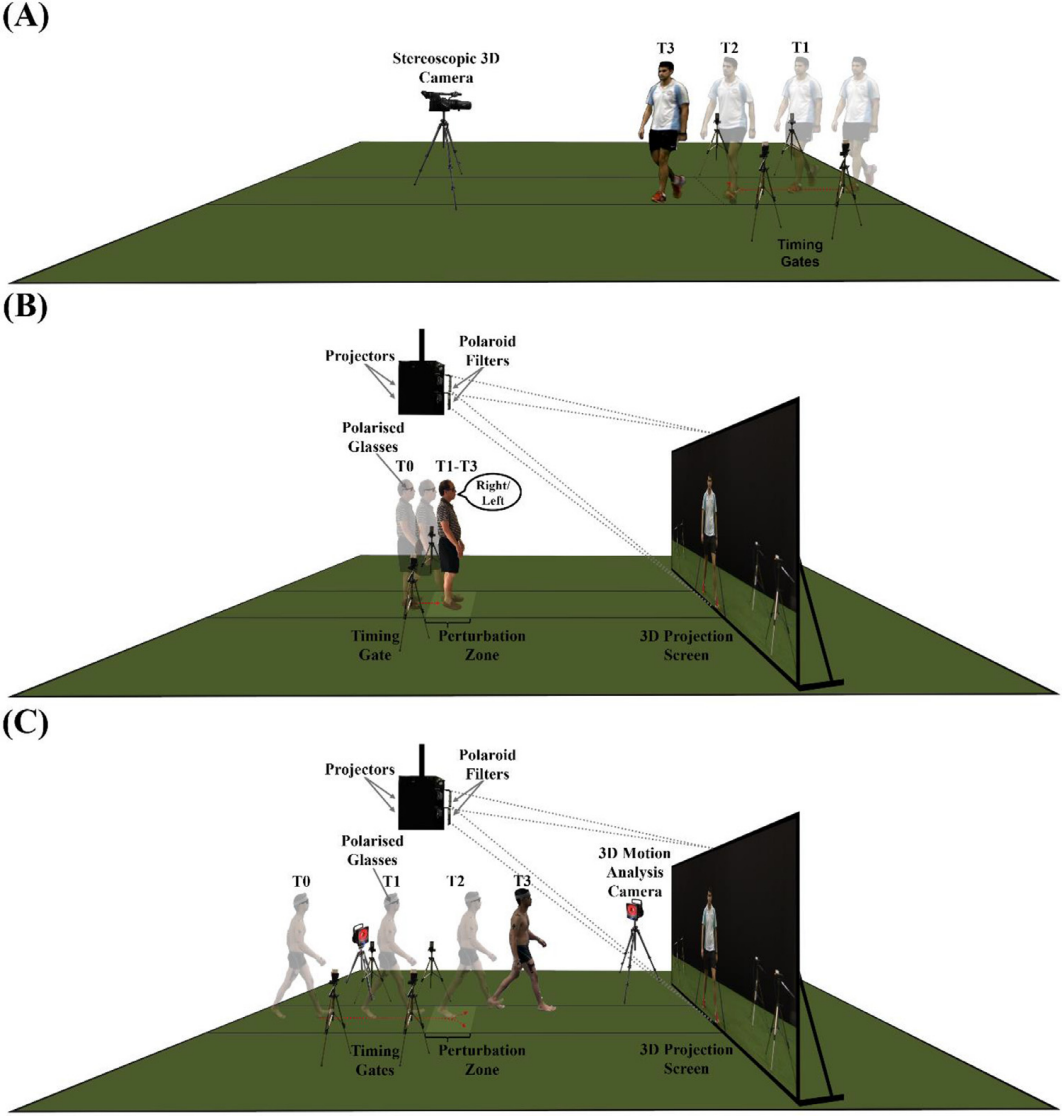
Geometrical considerations for developing the quasi-integrated stereoscopic three-dimensional (S3D) visual stimuli. This diagram illustrates the laboratory setup for (**A**) filming the S3D content, (**B**) verbal-perceptual task, and (**C**) steering-avoidance task. In (**A**), two sets of timing gates were used for determining the velocity of the oncoming actor. Only footages with the actor approaching at 1.50 ± 0.05 m/s were incorporated into the series of S3D stimuli. Common to both tasks illustrated in (**B)** and (**C)**, the perturbation zone where the participants would encounter the S3D person initiating a change in direction (i.e., unknown to them), was positioned in line with the placement of the S3D camera during filming to minimise inaccuracies in depth information. Abbreviations: 3D – three-dimensional, T0 – the time corresponding to the participants’ starting position, T1 – the time with which the S3D videos started playing, T2 – the time with which the S3D person initiated a change in direction to either side of the pathway (i.e., when the participants were within the perturbation zone), and T3 – the time corresponding to the end of the S3D videos. The illustrations were not drawn to scale.

The chosen scenarios for the S3D stimuli depicted an oncoming person approaching towards a participant while occupying the middle of the walkway (i.e., in the laboratory), either walking casually, or focused on texting with a mobile phone (Fig. 1A). A 25-year-old male actor was featured as the oncoming person in these scenarios. Both scenes featured the actor approaching at a velocity of 1.50 ± 0.05 m/s, for which this pace not only reflects that of a normative population based on the actor's age (i.e. between normal and fast gait) [16,17], but it is also in line with previous research [18] on perception of natural and fast human gait patterns in PwPD. Subsequently at a predetermined time, the actor would make a sudden change in direction, either to the left or the right of the walkway. As the S3D stimuli were designed for examining biological motion perception and unanticipated steering mechanics through a verbal-perceptual task (i.e., where participants would verbally indicating the direction they would take to avoid the oncoming actor; see Fig. 1B) and a steering-avoidance task (i.e., where participants would steer in the opposite direction and avoid the oncoming actor as quickly as they could while keeping within the pathway; see Fig. 1C) respectively, it was crucial to account for how participants would likely approach and react within the laboratory environment in order to create more quasi-realistic experiences.

To capitalize on the dual-purpose S3D stimuli, it was essential to set a common perturbation zone (i.e., unknown to the participants) for both the verbal-perceptual and steering-avoidance tasks, where the participants would approximately be at the same distance in front of the S3D person when the change in direction was initiated. The temporal precision required for both tasks could be achieved with the use of customised timing gates and an interface unit [10] that not only aid in triggering the video playback for both tasks (Fig. 1B and C), but also monitor and account for any inter-participant differences in walking velocity in the steering-avoidance task when positioned appropriately.

In addition to the temporal demands of the tasks, the scenarios filmed were designed to be quasi-immersed with the sports biomechanics laboratory at The University of Western Australia's School of Human Sciences (ethics approval: RA/4/5721). Thus, the prime consideration for developing the S3D stimuli was laboratory space. The positioning of the equipment used for filming and projecting the S3D footages, including the S3D camcorder (Panasonic AG-3DA1, Panasonic Corporation, Japan), the dual front projectors (Optoma EH2060, Coretronic Corporation, Taiwan; approximately 3.20 m above ground), and the projection screen (height x breadth: 2.36 m x 4.20 m), was relatively set due to their intrinsic parameters and/or dimensions (Fig. 1). The crux was to appropriately position these three pieces of equipment to ensure (i) sufficient space for filming the scenarios chosen and (ii) allow participants to approach and avoid the oncoming S3D person within the laboratory (Fig. 1A and Fig. 1C).

Another geometrical consideration was the spatial constraint imposed on the participants when avoiding the S3D person. Any lateral deviation away from the line of sight where the S3D camcorder was placed for filming would result in horizontal shearing of the S3D content when viewed. Hence, the width of the pathway was set at 1.20 m to minimize the effect of shearing as the participants deviate to either side, whilst ensuring sufficient space for two average-sized adults to walk side-by-side.

### Filming setup and techniques adopted for enhancing the participants’ overall visual experience

**Fig. 2:**
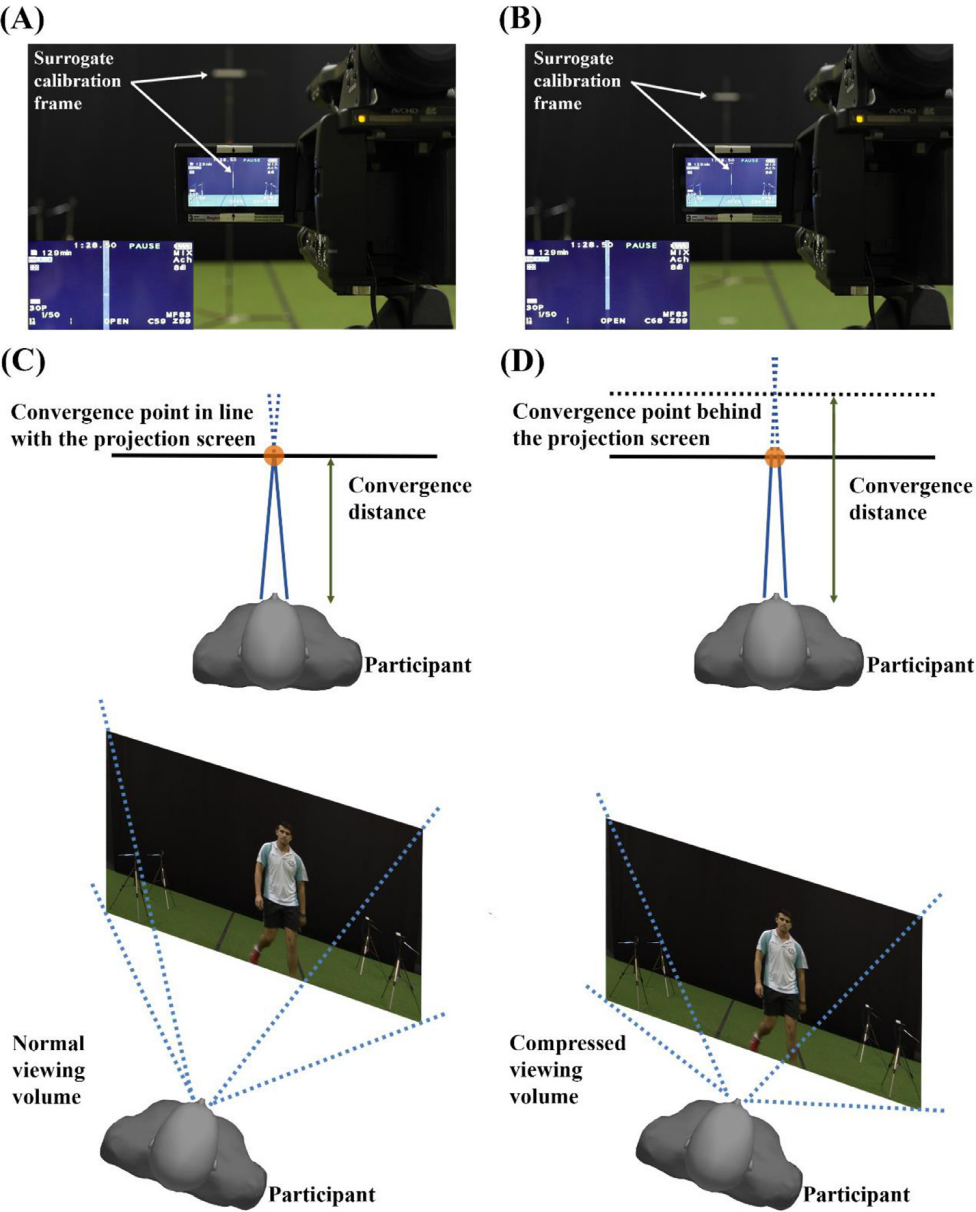
Scene calibrations and the effect of the convergence point adjustment. (**A**) A surrogate calibration frame for the projection screen was centralised and fitted within the stereoscopic three-dimensional (S3D) camcorder's field of view. The convergence point was initially adjusted in line with the calibration frame (as shown on the S3D camcorder's liquid-crystal display (LCD) monitor, the images from both lenses were interlaced). (**B**) The convergence point was readjusted and set behind the projection screen (see interlaced images on the S3D camcorder's LCD monitor). (**C**) A schematic illustration of a participant's viewing volume when observing a representative scene, with the convergence point in line with the projection plane. (**D**) A schematic representation of a participant's viewing volume being compressed when the convergence point was set behind the projection plane. The same scene would, therefore, appear closer to the observer due to the increased convergence distance.

Prior to filming, white balancing and brightness adjustments were carried out to ensure that the images recorded through both lenses of the S3D camcorder were matched. Additionally, the aspect ratios for both the S3D camcorder and the projection screen were 16:9. To retain the dimensions of the scene recorded when the S3D footages were being projected, a surrogate calibration frame for the projection screen (i.e., scaled to a height of 2.36 m) was positioned in the middle of the pathway and fitted within the S3D camcorder's field of view (Fig. 2A).

Unlike Lee et al. [8] who previously filmed their S3D footages with two standard video cameras at 25 Hz (in progressive mode, with Full High-Definition (HD) resolution at 1920 × 1080 pixel) from a position representative of their average participants’ height (1.70 m), all scenes in this study were filmed at 30 Hz (in progressive mode, with Full HD resolution) from a height of 1.45 m. At 1.70 m, the S3D camcorder had to be tilted downwards by 8° to fit the calibration frame within its field of view. Conversely, the scene would be angled upwards by 8° when projected onto the display screen, leading to discernible elevation and convergence of the pathway that degraded the quasi-immersive experience and realism of the visual stimuli.

Optic flow, as coined by Gibson [19], is purported to play a critical role in guiding goal-directed locomotor behaviours. An overt incongruity between the foreground (i.e., the laboratory flooring) and the background (i.e., the flooring of the filmed S3D stimuli) may not only disrupt the information flow through the participants’ eyes, but also influence the overall perceptual experience when engaging with the visual stimuli. Thus prior to determining the final filming position, rigorous filming was performed by varying the placement heights between 1.20 m and 1.60 m (with increments of 0.05 m). The scenes filmed were then visually inspected from various positions to simulate the viewing experience received by participants with different heights. While taking account into the limitations of vertical shearing and compression of the S3D contents as the participants approached towards the screen, the footages filmed from a height of 1.45 m delivered a better visual experience for participants with heights ranging from approximately 1.50 m to 1.85 m.

With the S3D camcorder set in place, the depth of the reference plane (i.e., convergence point) was subsequently adjusted. The convergence point would generally be set along the same plane as the projection screen to ensure the accuracy of depth information (Fig. 2A and C). The scenarios chosen for this research required the S3D person to make a direction change just before the projection plane. The legs and feet of the S3D person would, however, appear beyond the S3D camcorder's field of view when the manoeuvre was successfully completed in front of the projection plane. Due to the intrinsic properties of the S3D camcorder and the dimensions of the projection screen, the S3D person would be initiating the change in direction at approximately 5.30 m in front of the participants (i.e., within the perturbation zone where the S3D camera was placed during filming; see Fig. 1). When viewed from that distance, the lower limbs of the S3D person would appear relatively visible within the peripheral vision of any participant, thereby creating unrealistic visual experience for the participants when the lower limbs were beyond the screen.

A solution to this problem would be to compress the participants’ viewing volume so that the same scene would appear closer when viewed from the same position (Fig. 2D), thus allowing the lower limbs of the S3D person to appear less distinctive within the participants’ peripheral vision. This could be achieved by setting the convergence point behind the projection plane. In this study, the surrogate calibration frame was shifted backwards by 2.00 m, and the convergence point adjustment was attained by ensuring that the left and right images were interlaced along the new reference plane (Fig. 2B). Prior to establishing the position of the convergence point, rigorous filming was performed by varying the increases in convergence distance between 0.50 m and 2.50 m. The scenes filmed were subsequently displayed and viewed from varying heights between 1.50 m and 1.85 m within the perturbation zone to gauge the overall visual experience.

Lastly from a socio-psychological perspective, it was critical that the actor portrayed the appropriate body language and/or facial expressions while making a change in direction. With self-initiated changes in direction while walking either casually or focused on texting with a mobile phone, the actor often lacked the vital social cues when the pilot footages were reviewed. To enhance the realism of the scenarios filmed, appropriate visual cues were provided to guide the actor. Thus, the investigator approached towards the actor and initiated the change in direction at random behind the S3D camera while the actor reacted to the cues provided during the filming process.

### Post-processing of the filmed footages

Every scene recorded comprised two independent videos that were simultaneously captured through both lenses of the S3D camcorder. Each set of videos (i.e., left and right) was subsequently merged into a single 3840 × 1080-pixel resolution video file using Adobe After Effects CS6 software [20]. All videos were edited to exclude the actor's gait initiation phase to ensure that the actor was in constant motion once the videos began playing (i.e., T1 on Fig. 1). Regardless of the scenarios chosen, the time taken by the actor to approach and initiate the change in direction was standardized (i.e., T1 to T2 on Fig. 1A). Lastly, a frame of the empty background scene was also embedded to the start and the end of all trimmed footages to ensure a smoother transition between each trial.

### Passive Polaroid stereoscopic three-dimensional front projection system

**Fig. 3:**
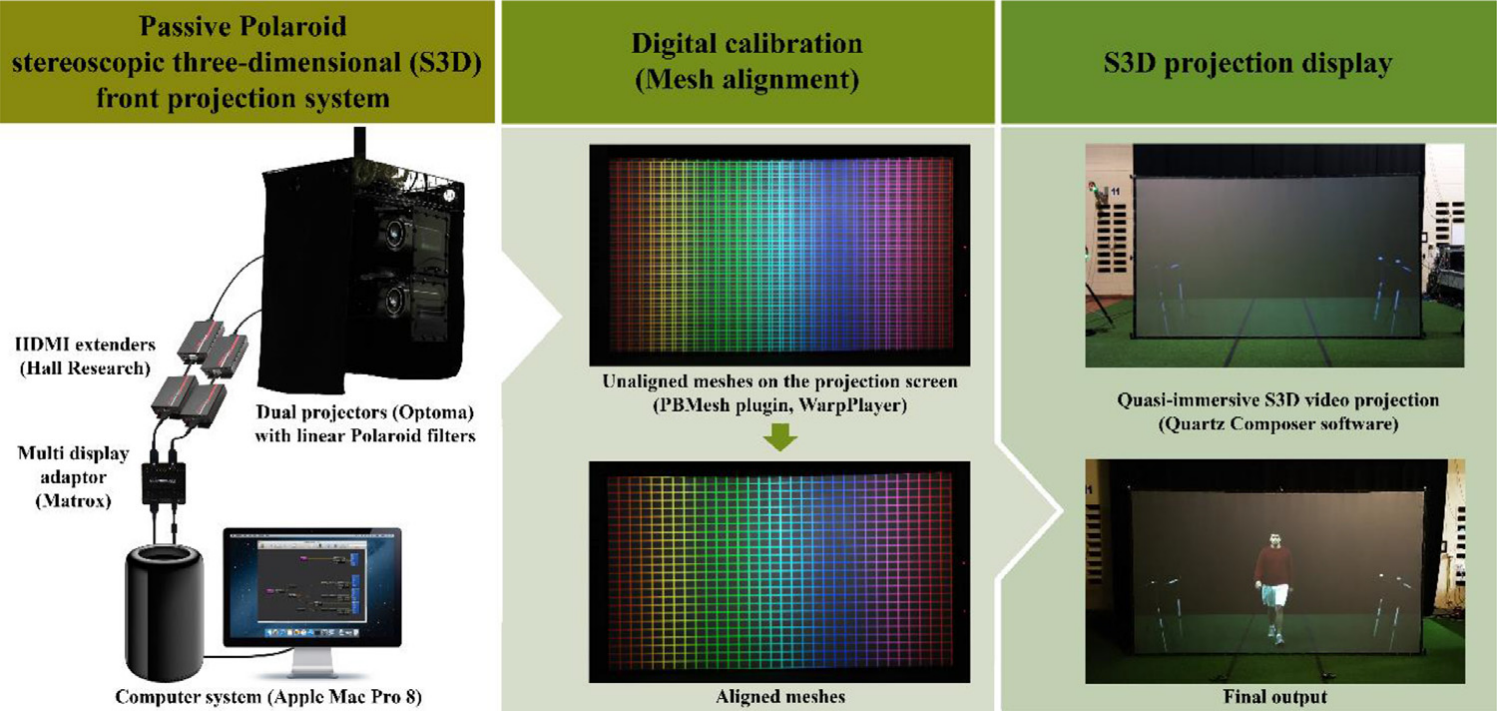
An overview of the passive Polaroid stereoscopic three-dimensional front projection system. Note: Both photos that display the S3D projections were taken under different lighting conditions. Abbreviations: HDMI ‒ High-Definition Multimedia Interface.

Following the editing process, a passive Polaroid S3D front projection system with dual projectors was used for presenting the videos (Fig. 3). Compared with rear projections [8], front projections are not only brighter but are also more space-saving (in terms of flooring). These were important considerations when investigating gait-related activities in a partially dimmed laboratory environment. In order for the projectors to display the video output from the computer system (Apple Mac Pro 8, 3.0 GHz 8-core Intel Xeon E5 and 16GB DDR3 memory) without any graphics compression over long distances, an external multi display adapter (Matrox Dualhead2go Digital Edition, Matrox Electronic Systems Ltd, Canada) and two sets of HD Multimedia Interface Extenders (Mini-Cat® brand UH-1BT, Hall Research, USA) were used (Fig. 3). This setup enabled each projector to present either the left or the right images from the processed videos.

**Fig. 4:**
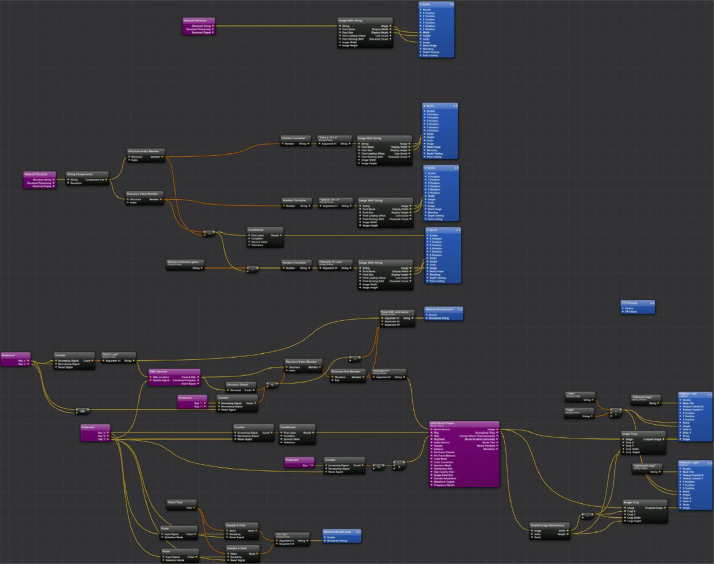
The customised composition in Quartz Composer Version 4.0 [24] that was used for stimuli projection and playback. Note: The composition was written by P.D. Bourke.

Prior to any video display, the projectors were digitally calibrated by warping and overlaying a mesh display from each projector onto the projection screen using Bourke's PBMesh plugin [21] and WarpPlayer software [22], see Fig. 3). This was performed to minimize the risk of visual fatigue (or eye strains) and depth field distortions from any projector misalignment [23]. Importantly, the use of digital calibration is a significant improvement from the work of Lee et al. [8] where the alignment of the projections were performed through physical adjustments of the projectors. The process is especially advantageous from a time perspective when the equipment used is often being shifted around in a shared laboratory space. Once the system was calibrated, the videos were presented using an improved, custom written program in Quartz Composer Version 4.0 [20] (Fig. 4). Specifically, this program consistently allowed every frame of the videos to be played unlike previous program by Lee et al. [8] where the video playback was incapable of beginning from the first frame. Importantly, this improvement not only ensured that all participants were consistently exposed to stimuli of the same duration, but also helped to improve the realism of the stimuli (i.e., the sudden appearance of the S3D person in motion and not from a ‘frozen’ pose).

To distinguish the left and right images from the screen, two complementary sets of orthogonally angled (± 45°) linear polarizing filters were required, with one pair each for the projectors and glasses to be worn by the participants. The Polaroid filters were appropriately angled to enable the viewer's left eye to receive the left images exclusively and vice versa for the right.

### Limitations

There were some limitations with the techniques and display technology described. By shifting the convergence point behind the projection plane, minor sacrifices in the accuracy of depth information were made to prioritize the realism of the scenarios featured in the S3D-BM visual stimuli. To minimize and ensure consistency in depth distortions across all participants, customised timing gates and an interface unit [8] were used to trigger the video playback so that the S3D person would initiate the change in direction at approximately the same distance in front of the participants (i.e., within the perturbation zone; Fig. 1B). Importantly, the positions of the timing gates that are used for triggering the video display could be adjusted appropriately to accommodate inter-participant differences in self-selected walking velocity. The distortions were, however, minimal with the static verbal-perceptual task (Fig. 1C).

## Conclusions

Striking an appropriate balance between experimental control and ecological validity is a quandary for researchers, especially in experiments that attempt to understand real-world behaviours. In order for laboratory-based investigations on biological motion perception and unanticipated steering mechanics to possess experimental control and ecological validity, we modified and improved Lee et al.’s [8] S3D cinematography techniques, software, and display technology to project semi-realistic ambulatory scenarios that are quasi-immersed with the laboratory environment. Despite the limitations, we have successfully created a series of twelve unique, yet similar S3D visual stimuli that are suitable for use in examining biological motion perception and unanticipated steering mechanics in PwPD. Importantly, this paper highlighted the necessary steps, theoretical and technical information to assist others by saving time in developing their S3D visual stimuli and display technology.

## Declaration of Competing Interest

None.
